# Supplementary material for: Durable superhydrophobic coatings for prevention of rain attenuation of 5G/weather radomes
Source: Nat Commun. 2023 May 19;14:2862. doi: 10.1038/s41467-023-38678-0 (PMC10198997; doi:10.1038/s41467-023-38678-0)
Supplement: Supplementary file 1 — SUPPLEMENTARY INFORMATION [file 41467_2023_38678_MOESM1_ESM.pdf]

Supplementary Information

Durable superhydrophobic coatings for prevention of rain attenuation of 5G/weather radomes

Jinfei Wei[1], Jiaojiao Zhang[1], Xiaojun Cao[3], Jinhui Huo[3], Xiaopeng Huang[1], and Junping Zhang[1, 2*]

[1]Center of Eco-Material and Green Chemistry, Lanzhou Institute of Chemical Physics, Chinese Academy of Sciences, 730000 Lanzhou, P.R. China

[2]Center of Materials Science and Optoelectronics Engineering, University of Chinese Academy of Sciences, 100049 Beijing, P. R. China

[3]Shandong Xinna Superhydrophobic New Materials Co. Ltd., 265402 Yantai, P.R. China

* Corresponding author: jpzhang@licp.cas.cn

This file includes:

Supplementary Methods

Supplementary Note

Supplementary Discussion

Supplementary Figures 1-25

Supplementary Tables 1-7

Supplementary References

Supplementary Methods

Materials

Hydrophilic silica nanoparticles (10-20 nm) were bought from Maikun Chemical Co., Ltd., China. TEOS (99.9%) and PFDTES (97%) were purchased from Gelest. POA (40 wt%) was supplied by Shandong Xinna Superhydrophobic New Material Co., Ltd., China. ABS plates (200 mm × 100 mm × 1 mm) were purchased from Shenzhen Shengyuanhua Engineering Plastic Materials Co., Ltd., China. Sodium methylsilicate was purchased from Jinan Silicon Port Chemical Co., Ltd., China. Anhydrous ethanol (99.7 wt%), butyl acetate (99 wt%) and other reagents were bought from China National Medicines Co., Ltd. All the chemical reagents were used as received without further purification. The 5G radomes were supplied by Guangdong Shenglu Communication Technology Co., Ltd. and Shenzhen Weijing High-tech Materials Technology Co., Ltd., China. The weather radomes were supplied by local meteorological departments.

Dynamic superhydrophobicity tests

(1) Water droplet impacting. A 10 μL water droplet was released from 1 cm height to impact on the coating. The bounce times, solid-liquid contact time and bounce height in the first impact/bounce cycle were recorded using a high-speed video camera (FASTCAM, Mini UX100) at 4000 fps.

(2) High-pressure water jetting. The coating was placed on a 45° tilted platform and scoured by a high-pressure water jet (50 kPa) for a period. The distance between the coating and the water outlet is 20 cm. At certain time intervals, the CA and SA were measured.

(3) Simulated rainfall. We used a shower with a nozzle diameter of 0.4 mm to form simulated rainfall with a flow of ~3.2 mL/s (~13 drops per second). The 15° tilted coating was placed 45 cm beneath the shower. At certain time intervals, the CA and SA were measured.

Mechanical robustness tests

(1) Reciprocating abrasion. The coating was placed face-down to sandpaper (1000 meshes), and then horizontally moved along the sandpaper for several cycles (40 cm per cycle) under a pressure of 2.3 kPa. After certain cycles, the CA and SA were measured.

(2) Taber abrasion (ASTM D4060). A Taber type abrasion tester (Dongguan Yaoke Instrument Equipment Co., Ltd.) with CS 10 grinding wheel was employed and the load is 125 g. After certain cycles, the CA and SA were measured.

(3) Tape-peeling (ASTM D3359). The 3M tape (20 mm in width and 0.6 mm in thickness) with an adhesion strength of 3000 N m^{-1} to standard stainless steel was used. The 3M tape was pressed onto the surface of the coating using a cylindrical copper block (2.3 kPa). Then, the tape was peeled off, and the CA and SA of the coatings were measured.

Weather resistance tests

(1) UV aging (GB/T 14522-2008). The coating was placed horizontally in the UV accelerated weathering tester (ZN-P, Xinlang, Shanghai, China) with eight UV-B bulbs (280-315 nm, 40 W), The coating was treated for several cycles with 4 h UV irradiation at 60 °C and 4 h condensation at 50 °C in each cycle. After certain cycles, the CA and SA were measured.

(2) Corrosion resistance (ASTM B117). The corrosion resistance of the coating was investigated by the neutral salt spray test. The coating was 45° inclined in the chamber of a salt spray corrosion tester (Shanghai Guangpin Testing Equipment Manufacturing Co., Ltd., China) and continuously exposed to the 5 wt% NaCl salt spray at 35 °C. The corrosion resistance of the coating was also investigated by immersion in 1 M $HCl_{(aq)}$ and 1 M $NaCl_{(aq)}$ solutions for a period. At certain time intervals, the CA and SA were measured.

(3) Thermal stability (GB/T 2423.2-2008, GB/T 2423.1-2008). The coating was placed horizontally in an oven at 150 °C or in a refrigerator at -18°C for a period. At certain time intervals, the CA and SA were measured.

(4) Long-term outdoor weather resistance (ASTM D1014). The coatings were 45° inclined in the outdoor environment in Yantai, Shandong, P.R. China for two years to evaluate the long-term outdoor weather resistance. At certain time intervals, the CA and SA were measured.

Evaluation of preventing rain attenuation performance

A custom-made setup was used for evaluating performance of the coating in preventing rain attenuation of 5G radomes. The setup mainly includes a signal source, a signal transmitter, a signal receiver, a spectrograph, and a RF cable (Supplementary Fig. 21). The setup has good repeatability as verified by testing signal transmission without placing the radome between the signal transmitter and the signal receiver (Supplementary Fig. 22). The specific method for evaluating performance of the coating in preventing rain attenuation is as follows. First, a signal value (named Signal 1) without placing a radome between the signal transmitter and the signal receiver was recorded. Then, a radome was put between the signal transmitter and the signal receiver and the signal value was recorded (named Signal 2). The signal loss caused by the radome can be calculated as follows:

$$\text{Signal loss} = \text{Signal 2} - \text{Signal 1} \quad (1)$$

The signal losses of the blank radome and the coated radome before and after 48 h simulated rainfall were recorded to evaluate performance of the coating in preventing rain attenuation. The signals of four 5G millimeter-wave frequency bands (N257, N258, N260 and N261) at low, middle, and high frequency points were tested. The sources of error for the repeatability measurements are mainly the sensitivity of the test instrument and verticality between the radomes and the signal transmitter & signal receiver.

Anti-icing performance tests

The anti-icing performance of samples was evaluated using a custom-made ice adhesion device. The sample was placed on a horizontal cooling plate with a temperature of -20 °C. The temperature in the chamber was controlled at -5 °C using a thermostatic ethanol bath and the relative humidity was controlled at ~97% using a mixture of wet and dry N_2. The water freezing process (60 μL deionized water) was recorded by a CCD camera to measure the water freezing time. For the ice adhesion strength test, 1.0 mL of deionized water was injected into a glass column on the sample surface and was frozen completely for 3 h. Then, a gauge was driven at a constant speed by the motorized stage to push the icicle. The push force (F) for separating the icicle from the sample surface was recorded. The contact area (A) between the icicle and the

sample surface is 100 mm^2. So, the ice adhesion strength (τ) can be calculated according to formula (2).

$$\tau = F / A \tag{2}$$

Characterization

A field emission SEM (JSM-6701F, JEOL) was used to capture micrographs of the coatings. Before SEM observation, the samples were fixed on copper stubs using conductive tape and coated with a layer of gold film (ca. 7 nm in thickness). The EDS spectra and surface elemental mapping of the samples was carried out using the energy dispersive spectrometer attached to the SEM. The micrographs of samples were taken using a field emission transmitting electron microscope (TEM, TECNAI-G2-F30, FEI). The surface chemical composition of the coating was studied by XPS using a VG ESCALAB 250 Xi spectrometer equipped with a monochromatized Al Kα X-ray radiation source and a hemispherical electron analyzer. The FTIR spectrum of the sample was recorded on the Thermo Nicolet Nexus spectrophotometer (Thermo, Madison, USA) in 4000-400 cm^{-1} using KBr pellets. The CA and SA on the coatings were measured with 10 μL droplets using a contact angle system OCA20 (Dataphysics, Germany), and a minimum of five positions were measured. The dielectric strength of the coating was tested using a breakdown voltage tester (DJC-50KV) according to IEC 60243-1:2013. The thicknesses of the coatings were recorded using an electronic digital display micrometer with a resolution of 1 μm (SYA1704569, SYNTEK). The thicknesses of the coatings were calculated according to formula (3).

$$\text{Coating thickness} = T_1 - T_2 \tag{3}$$

where T_1 is the thickness of the substrate with the coating and T_2 is the thickness of the substrate.

Supplementary Note

Design of superhydrophobic coatings with high dynamic superhydrophobicity. Superhydrophobicity of a surface is positively correlated with tiers of its hierarchical structure.[1] The more the tiers, the better the superhydrophobicity is. Also, dynamic superhydrophobicity depends on the hammer pressure (P_h) of droplets and the capillary pressure (P_c) generated within the surface structure.[2] For droplets impacting a horizontal surface, P_h can be estimated using formula (4):[2]

$$P_h \approx 0.2\rho C v \quad (4)$$

Here, ρ is water density, C is sound velocity (C_{water} = 1497 m s^{-1}), and v is impact velocity.

P_c can be estimated using formula (5):[2]

$$P_c \approx 2\gamma r \sin^2(\frac{\theta_{adv}}{2})/d^2 \quad (5)$$

Here, d is the mean distance between protrusions, r is the radius of the constituting particles, γ is surface tension of the liquid, and θ_{adv} is the advancing CA_{water} on a smooth fluorosilane coated substrate.

According to the formulas, penetration of impacting droplets can be effectively inhibited when $P_c > P_h$. Also, the smaller the d, the larger the P_c is, which means a dense surface structure can enhance dynamic superhydrophobicity.

Supplementary Discussion

The effects of the mass ratio of POA to fluoroPOS@silica nanoparticles on the coating performance are shown in Supplementary Fig. 4. The increase in the mass ratio from 0.2:1 to 0.6:1 had no obvious influence on CA and SA of the coating but reduced the coating thickness from 134.4 ± 2.9 μm to 68.2 ± 4.1 μm.

When the mass ratio was 0.2:1, the superhydrophobicity of the coating only slightly decreased during 180 s high-pressure water jetting at 50 kPa. With increase of the mass ratio, the impalement resistance gradually decreased, as more adhesive reduced stability of the Cassie-Baxter state.[3] When the mass ratio was 0.6:1, the superhydrophobicity decreased obviously during only 30 s high-pressure water jetting.

Regarding changes in the CA and SA during the reciprocating abrasion test, the mechanical robustness of the coatings was first enhanced with increasing the mass ratio to 0.4:1, and then reduced with further increasing the mass ratio to 0.6:1. When the POA content is insufficient (mass ratio <0.4:1), the linkages among the particles in the coating and the adhesion between the coating and the substrate are weak, and thus the coatings can be easily damaged during abrasion. When the POA content is excess (mass ratio >0.4:1), more POA are exposed during abrasion, and thus the superhydrophobicity also declines.

Considering both impalement resistance and mechanical robustness, the POA/fluoroPOS@silica coating with a mass ratio of 0.4:1 was used for further studies.

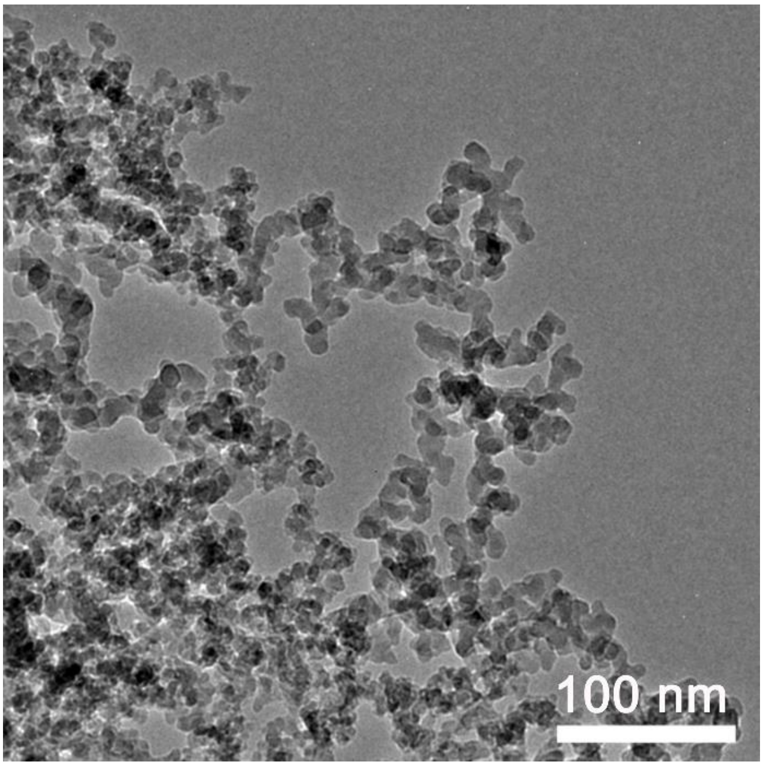

Supplementary Fig. 1 TEM image of hydrophilic silica nanoparticles.

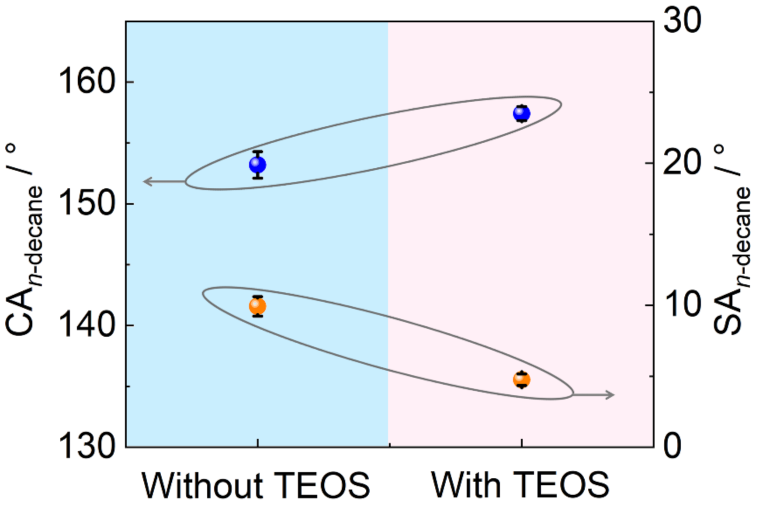

Supplementary Fig. 2 Effect of TEOS on CA$_{n\text{-decane}}$ and SA$_{n\text{-decane}}$ of the fluoroPOS@silica coatings without POA. Data are shown as mean ± SD, n = 5.

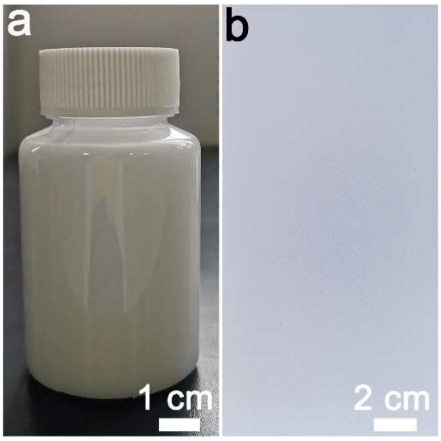

Supplementary Fig. 3 Photographs of the a POA/fluoroPOS@silica suspension and b POA/fluoroPOS@silica coating.

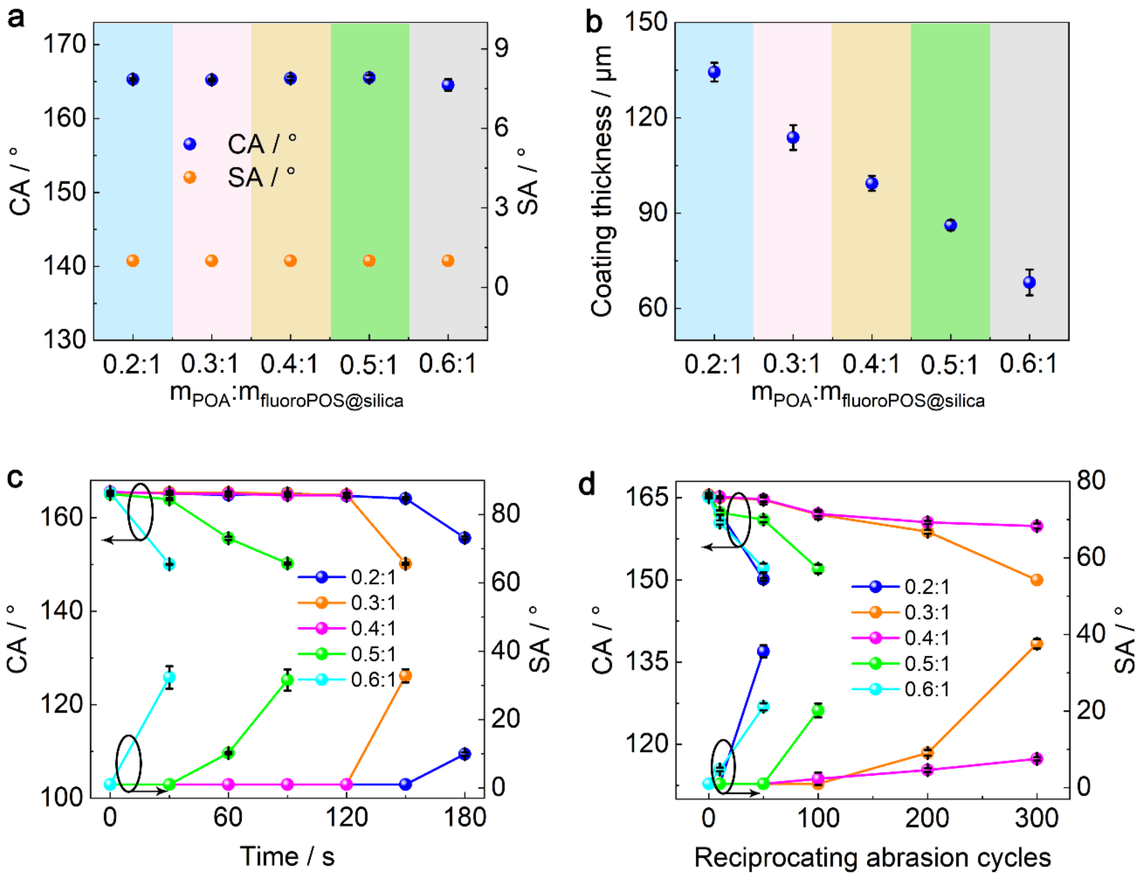

Supplementary Fig. 4 Effects of the mass ratio of POA to fluoroPOS@silica nanoparticles on comprehensive performance of the POA/fluoroPOS@silica coatings. Effects of the mass ratio of POA to fluoroPOS@silica nanoparticles on **a** superhydrophobicity, **b** coating thickness, **c** impalement resistance (high-pressure water jetting at 50 kPa) and **d** mechanical robustness of the POA/fluoroPOS@silica coatings. Data are shown as mean ± SD, n = 5.

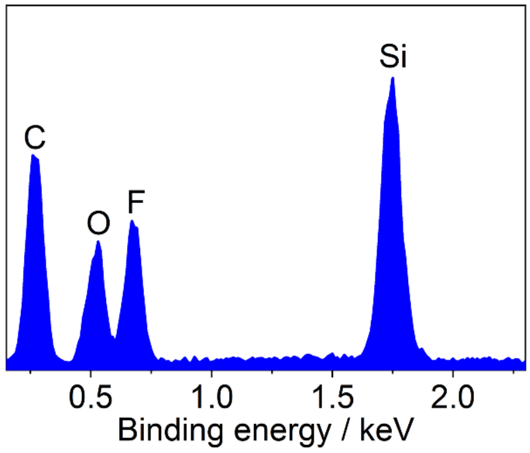

Supplementary Fig. 5 EDS spectrum of the POA/fluoroPOS@silica coating.

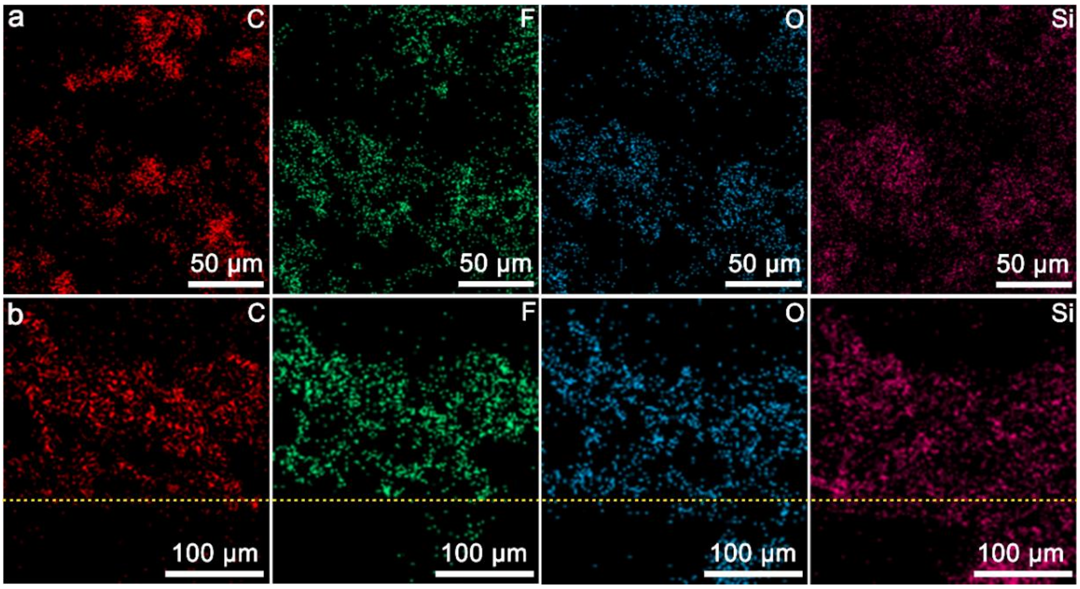

Supplementary Fig. 6 EDS elemental maps of the POA/fluoroPOS@silica coating. a Surface and **b** cross-sectional EDS elemental maps of the POA/fluoroPOS@silica coating.

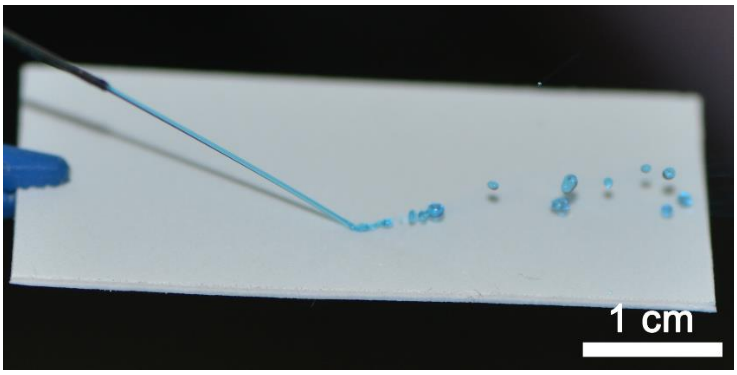

Supplementary Fig. 7 Photograph of the POA/fluoroPOS@silica coating with a water jet (dyed with methylene blue) bouncing off.

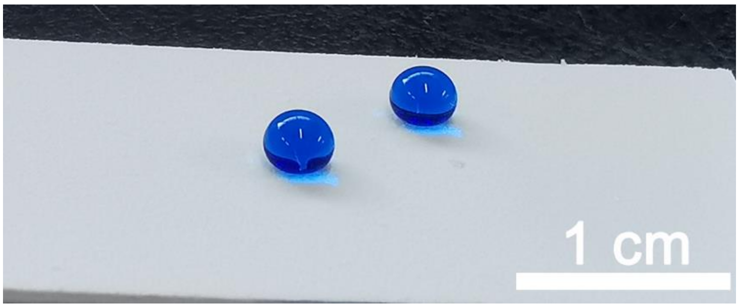

Supplementary Fig. 8 Photograph of the POA/fluoroPOS@silica coating with water droplets (dyed with methylene blue) after 120 s high-pressure water jetting at 50 kPa.

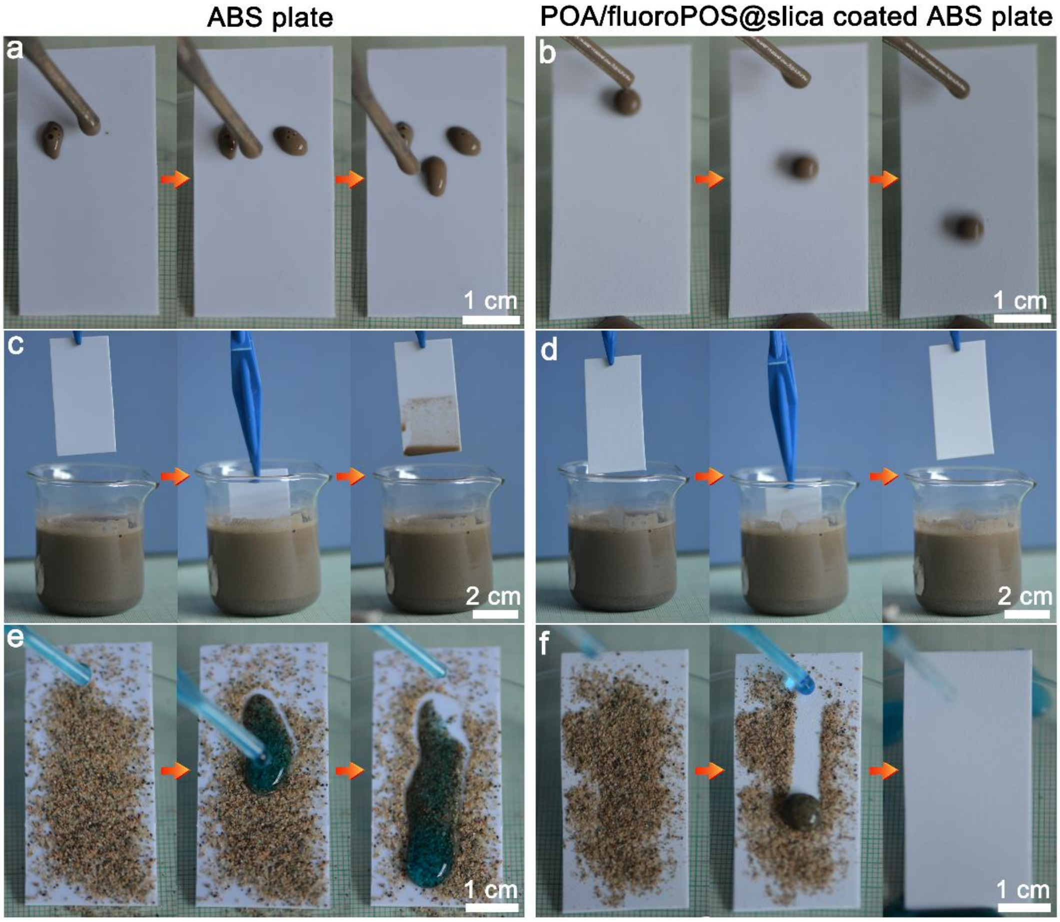

Supplementary Fig. 9 Anti-fouling and self-cleaning properties of the POA/fluoroPOS@silica coatings. Dropping sewage on the **a** ABS plate and **b** POA/fluoroPOS@silica coated ABS plate. Immersing the **c** ABS plate and **d** coated ABS plate in sewage. Removing sand particles on the **e** ABS plate and **f** coated ABS plate by water droplets (dyed with methylene blue).

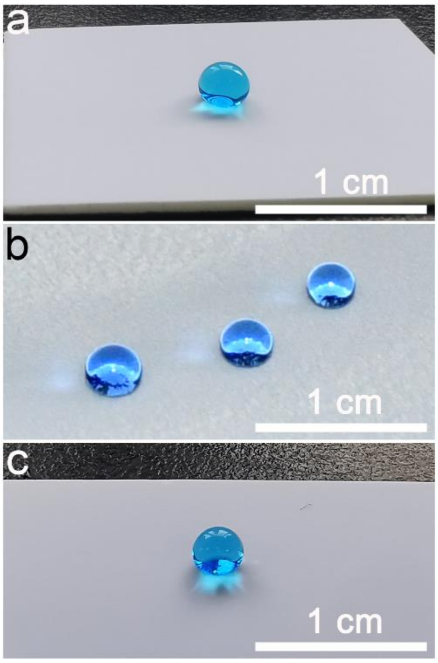

Supplementary Fig. 10 Photographs of the POA/fluoroPOS@silica coating with water droplets after mechanical robustness tests. Photographs of the POA/fluoroPOS@silica coating with water droplets (dyed with methylene blue) after **a** 300 cycles reciprocating abrasion, **b** 50 cycles Taber abrasion or **c** 75 cycles tape-peeling.

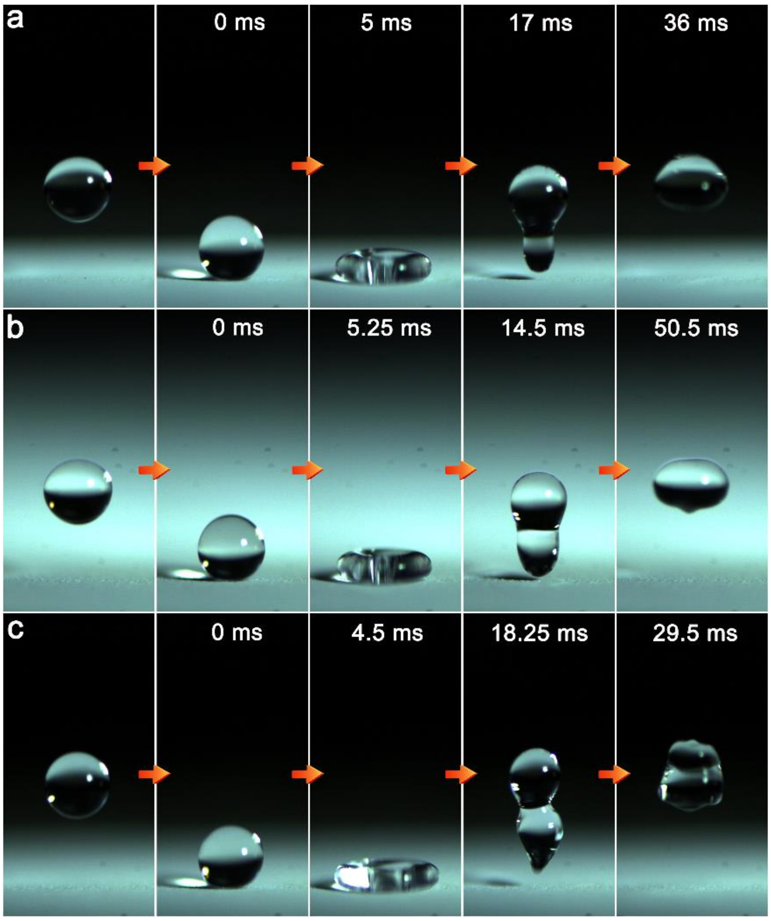

Supplementary Fig. 11 Impact/bounce of water droplets on the POA/fluoroPOS@silica coating after mechanical robustness tests. Snapshots of impact/bounce of water droplets released from 10 mm height on the POA/fluoroPOS@silica coating after **a** 300 cycles reciprocating abrasion, **b** 50 cycles Taber abrasion or **c** 75 cycles tape-peeling.

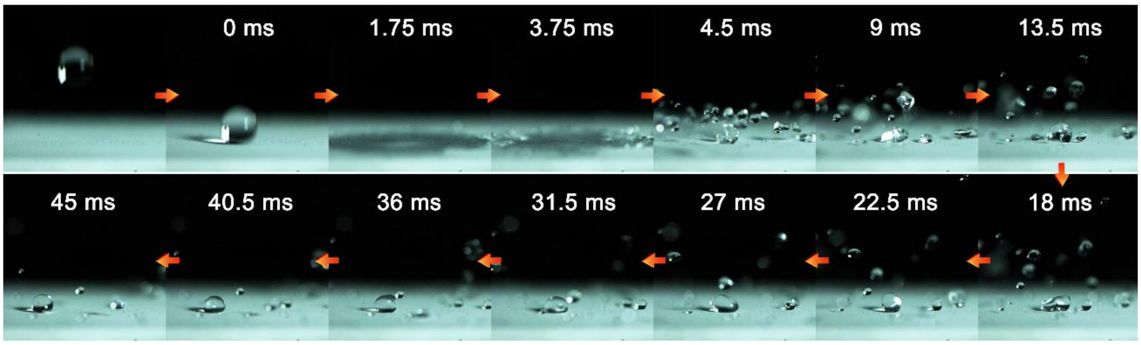

Supplementary Fig. 12 Snapshots of impact/bounce of a water droplet released from 100 cm height on the POA/fluoroPOS@silica coating after 300 cycles reciprocating abrasion.

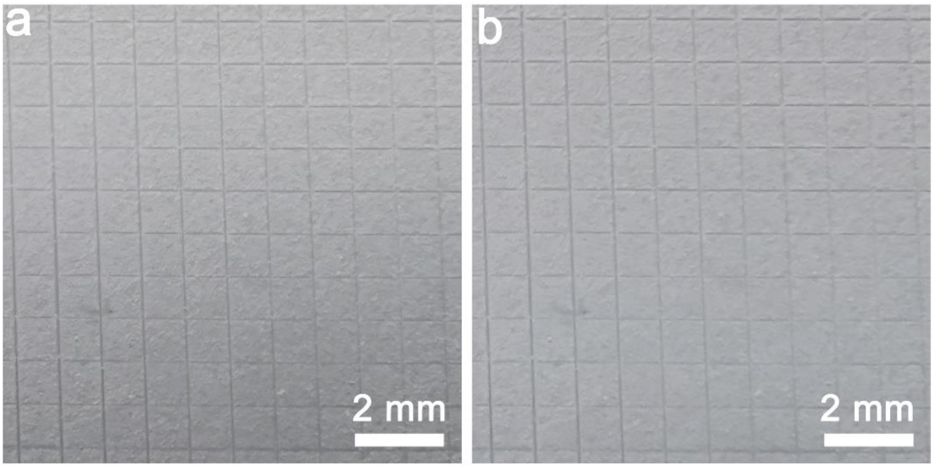

Supplementary Fig. 13 Adhesion strength of the POA/fluoroPOS@silica coating on the ABS plate. Photographs of the POA/fluoroPOS@silica coating **a** before and **b** after the hundred-grid adhesion strength test (ASTM D3359). Briefly, regular grids with an interval of 1 mm were formed using a hundred-grid knife on the coating. Then, the 3M tape was pressed onto the surface of the coating under a load of 2 kg and then peeled off. The intactness of the coating was checked.

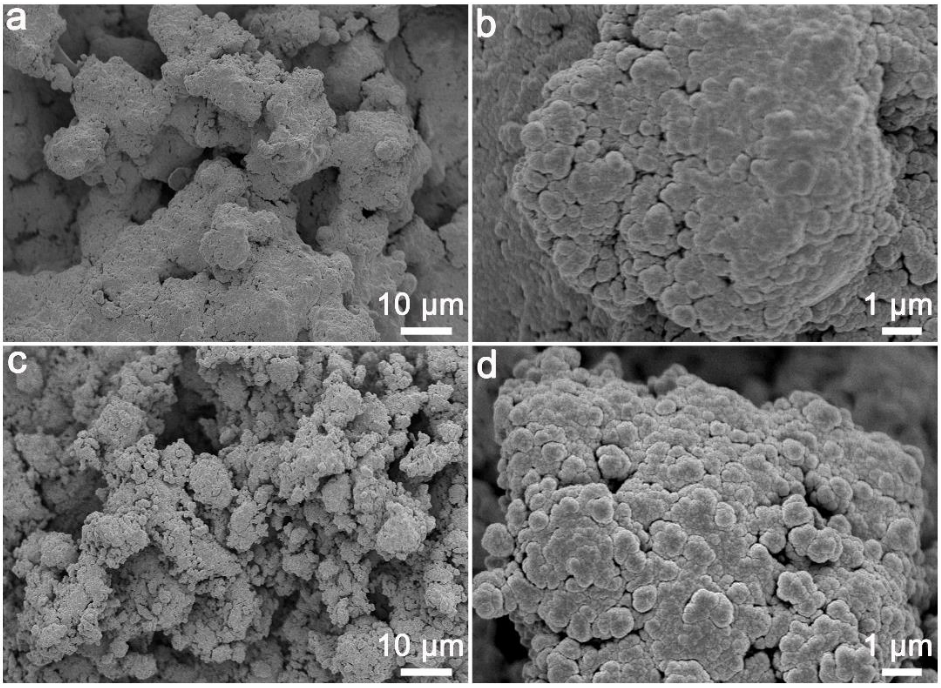

Supplementary Fig. 14 SEM images of the POA/fluoroPOS@silica coating after mechanical robustness tests. SEM images of the POA/fluoroPOS@silica coating after **a, b** 50 cycles Taber abrasion or **c, d** 75 cycles tape-peeling.

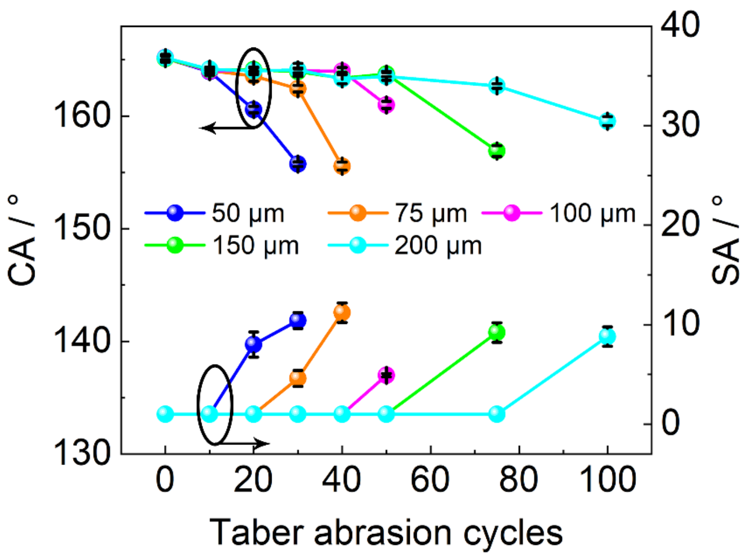

Supplementary Fig. 15 Effect of coating thickness on mechanical robustness. Changes in CA and SA of the POA/fluoroPOS@silica coatings with different thickness during the Taber abrasion test. The data are shown as mean ± SD, n = 5.

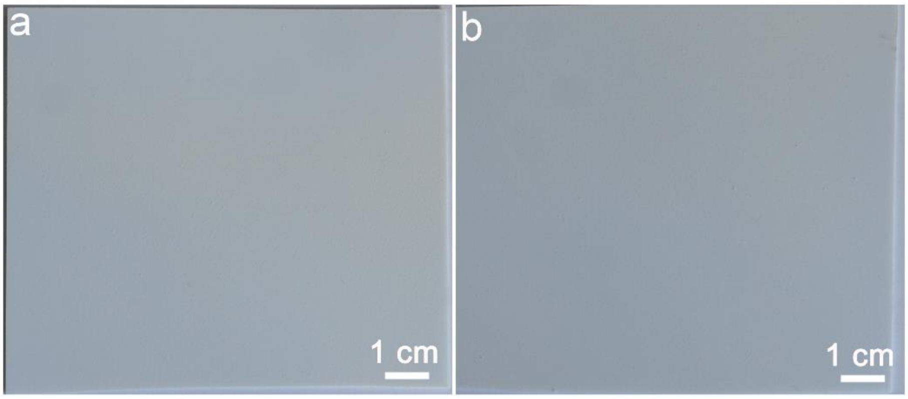

Supplementary Fig. 16 Photographs of the POA/fluoroPOS@silica coating after weather resistance tests. Photographs of the POA/fluoroPOS@silica coating after **a** 30 cycles of the UV ageing test and **b** 840 h of the neutral salt spray test.

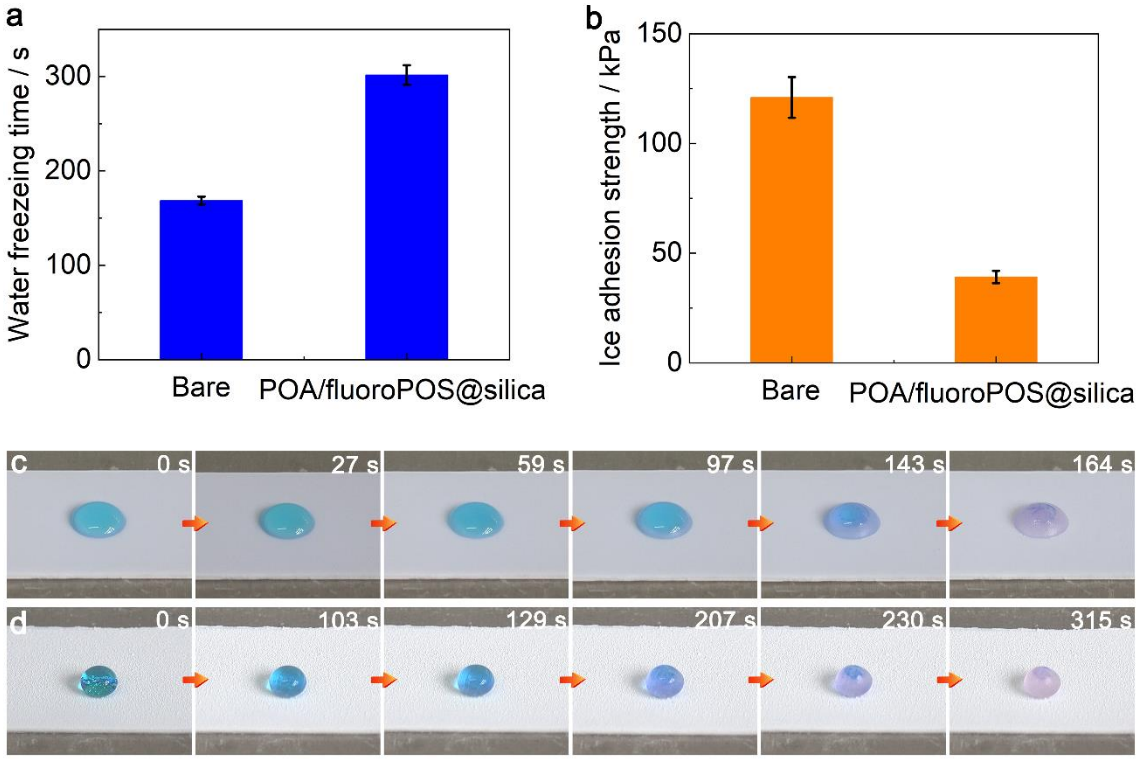

Supplementary Fig. 17 Anti-icing performance of the POA/fluoroPOS@silica coating. a Water freezing time (60 μL) and **b** ice adhesion strength on the ABS plate and POA/fluoroPOS@silica coated ABS plate. Freezing process of water droplets (60 μL) on the **c** ABS plate and **d** POA/fluoroPOS@silica coated ABS plate. Data in **a** and **b** are shown as mean ± SD, n = 3.

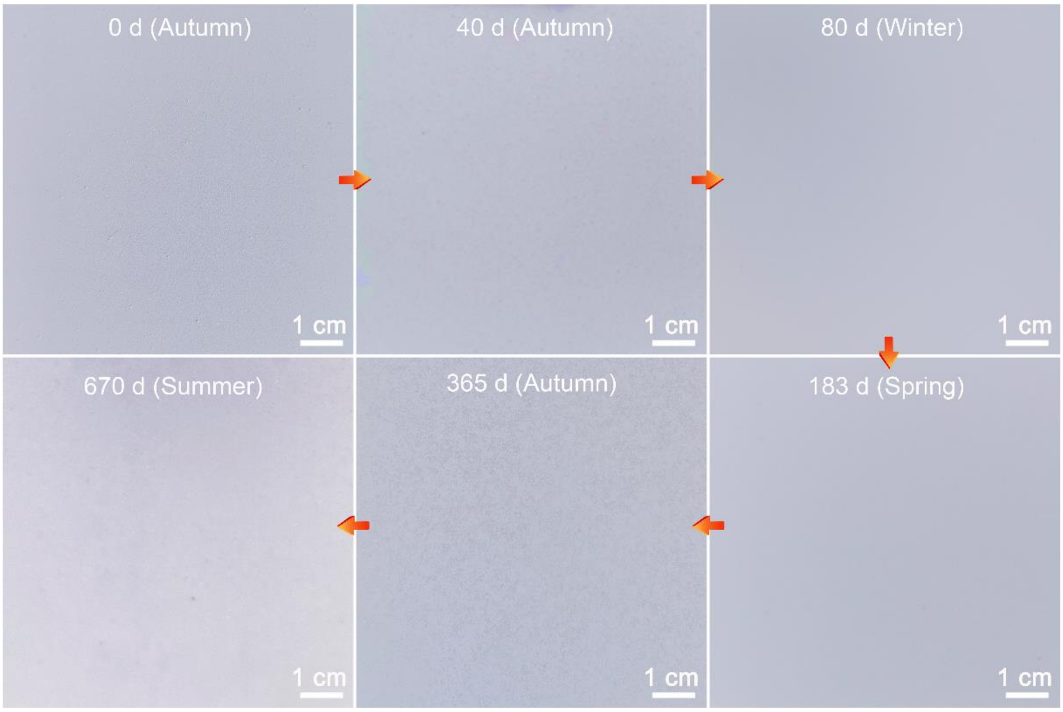

Supplementary Fig. 18 Photographs of the POA/fluoroPOS@silica coating during the weather resistance test in the outdoor environment.

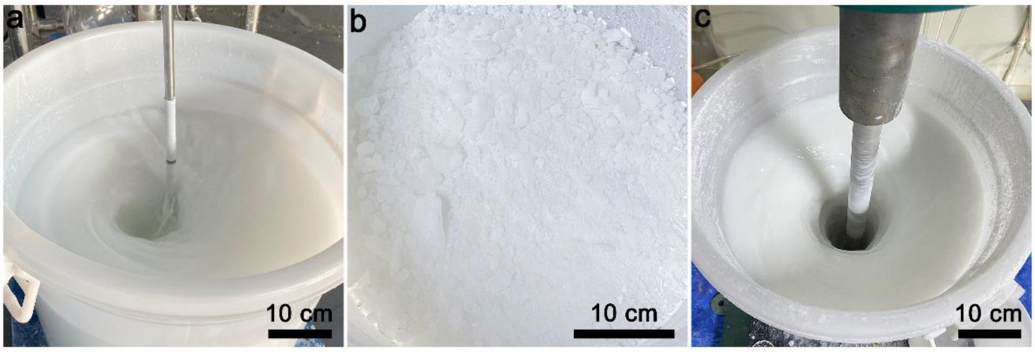

Supplementary Fig. 19 Large-scale preparation. Large-scale preparation of the **a** fluoroPOS@silica suspension, **b** fluoroPOS@silica nanoparticles containing ethanol and **c** POA/fluoroPOS@silica suspension.

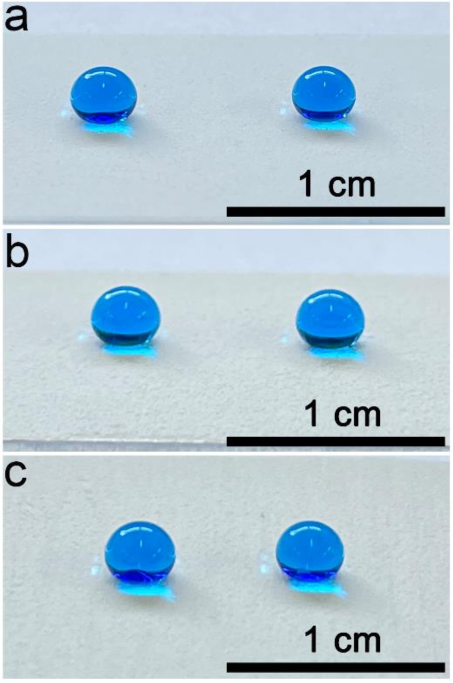

Supplementary Fig. 20 Superhydrophobicity of the POA/fluoroPOS@silica coatings on various substrates. Photographs of water droplets (dyed with methylene blue) on the surfaces of **a** glass, **b** Al alloy and **c** stainless steel with the POA/fluoroPOS@silica coating.

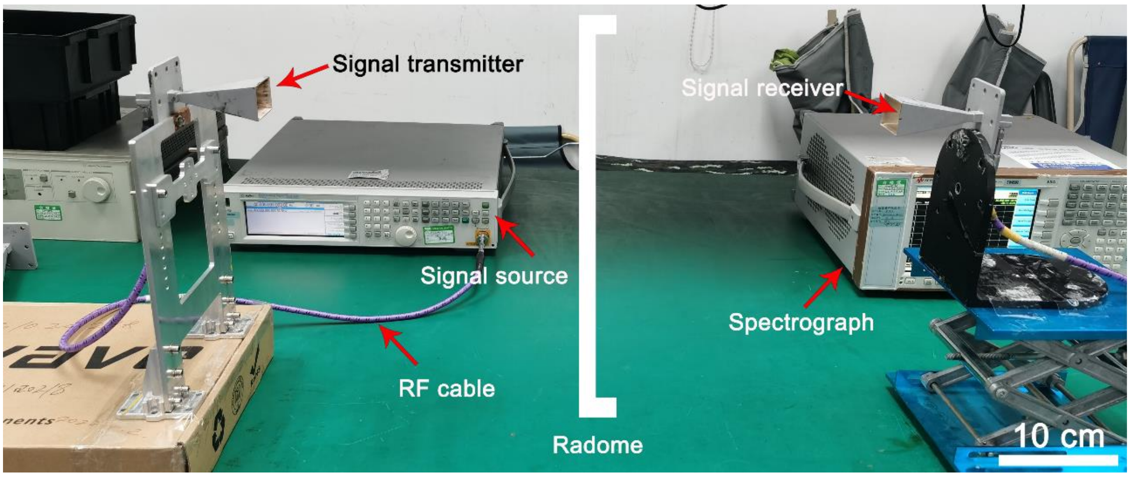

Supplementary Fig. 21 Photograph of the setup for measuring the rain attenuation performance of 5G radomes.

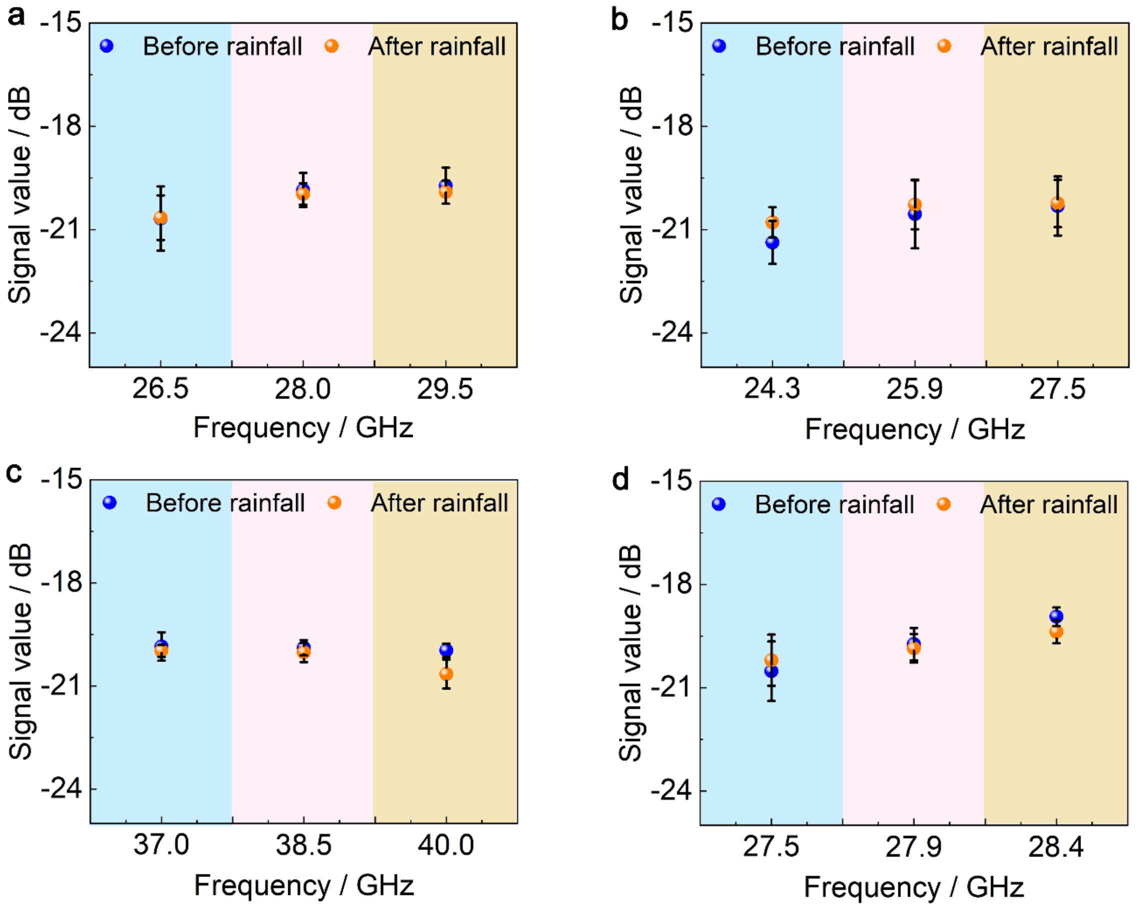

Supplementary Fig. 22 Repeatability of the setup for measuring the rain attenuation performance of 5G radomes. Signal transmission of the **a** N257, **b** N258, **c** N260 and **d** N261 5G millimeter-wave frequency bands without radomes before and after 48 h simulated rainfall. Data are shown as mean ± SD, n = 6.

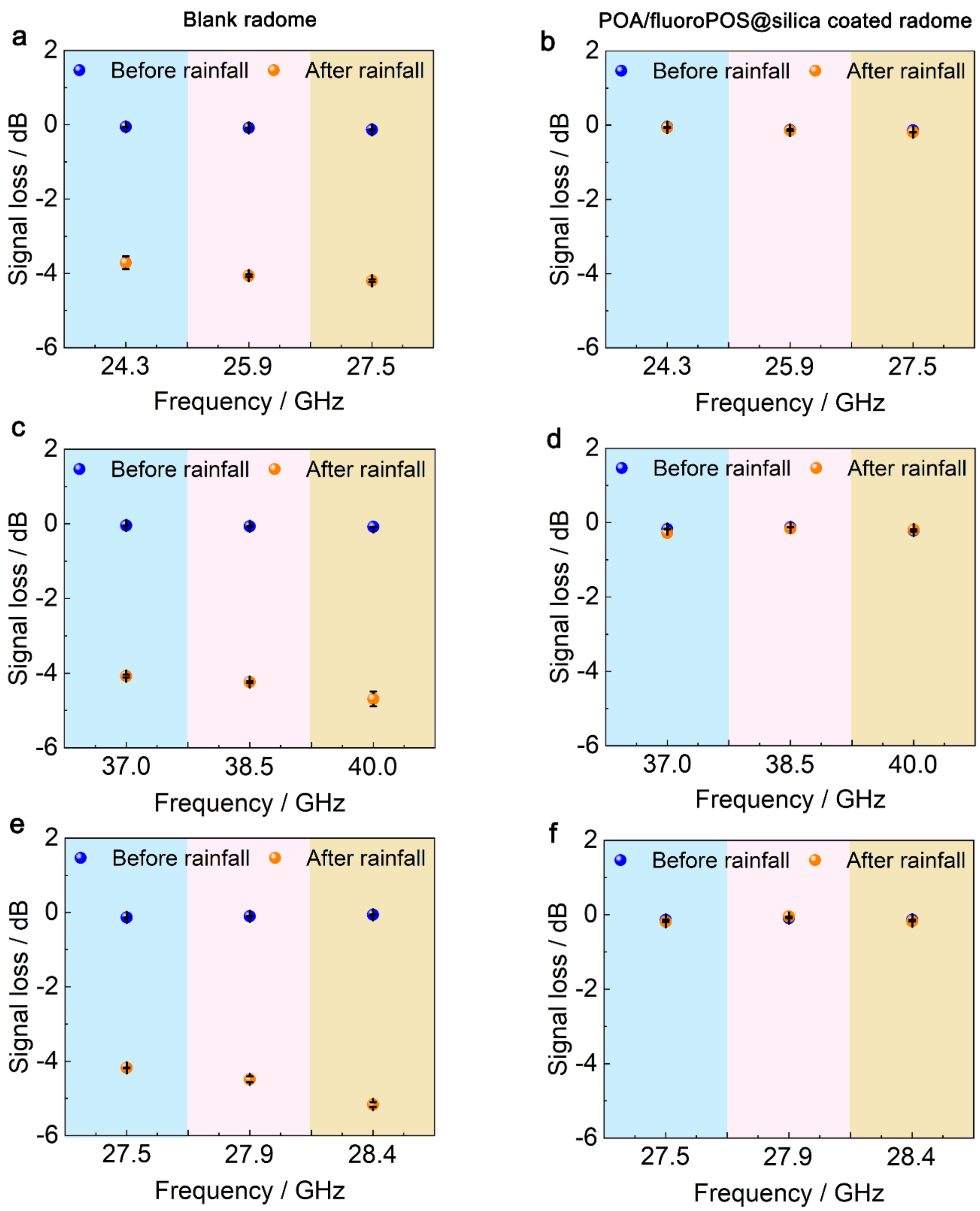

Supplementary Fig. 23 Preventing rain attenuation of 5G radomes by the POA/fluoroPOS@silica coating. Signal loss of the **a, b** N258, **c, d** N260 and **e, f** N261 5G millimeter-wave frequency bands through the blank and POA/fluoroPOS@silica coated 5G radomes before and after 48 h simulated rainfall. Data are shown as mean ± SD, n = 3.

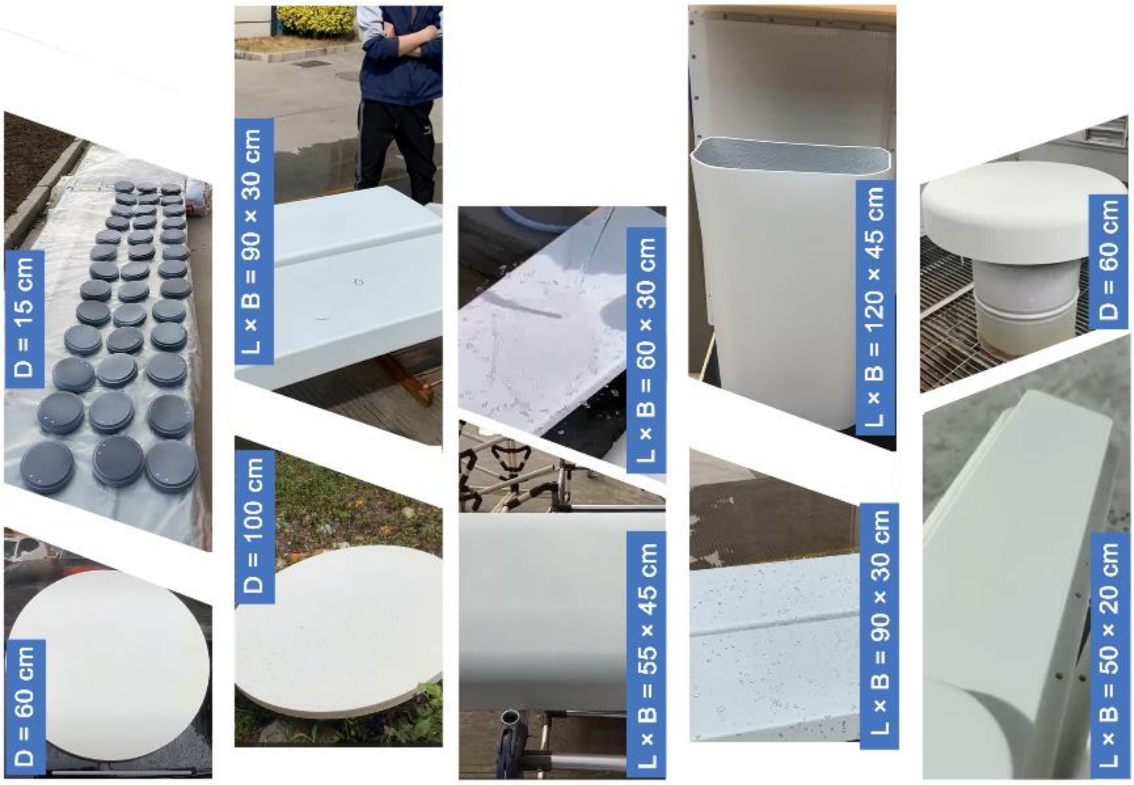

Supplementary Fig. 24 Photographs of the 5G radomes in different shapes with the POA/fluoroPOS@silica coating.

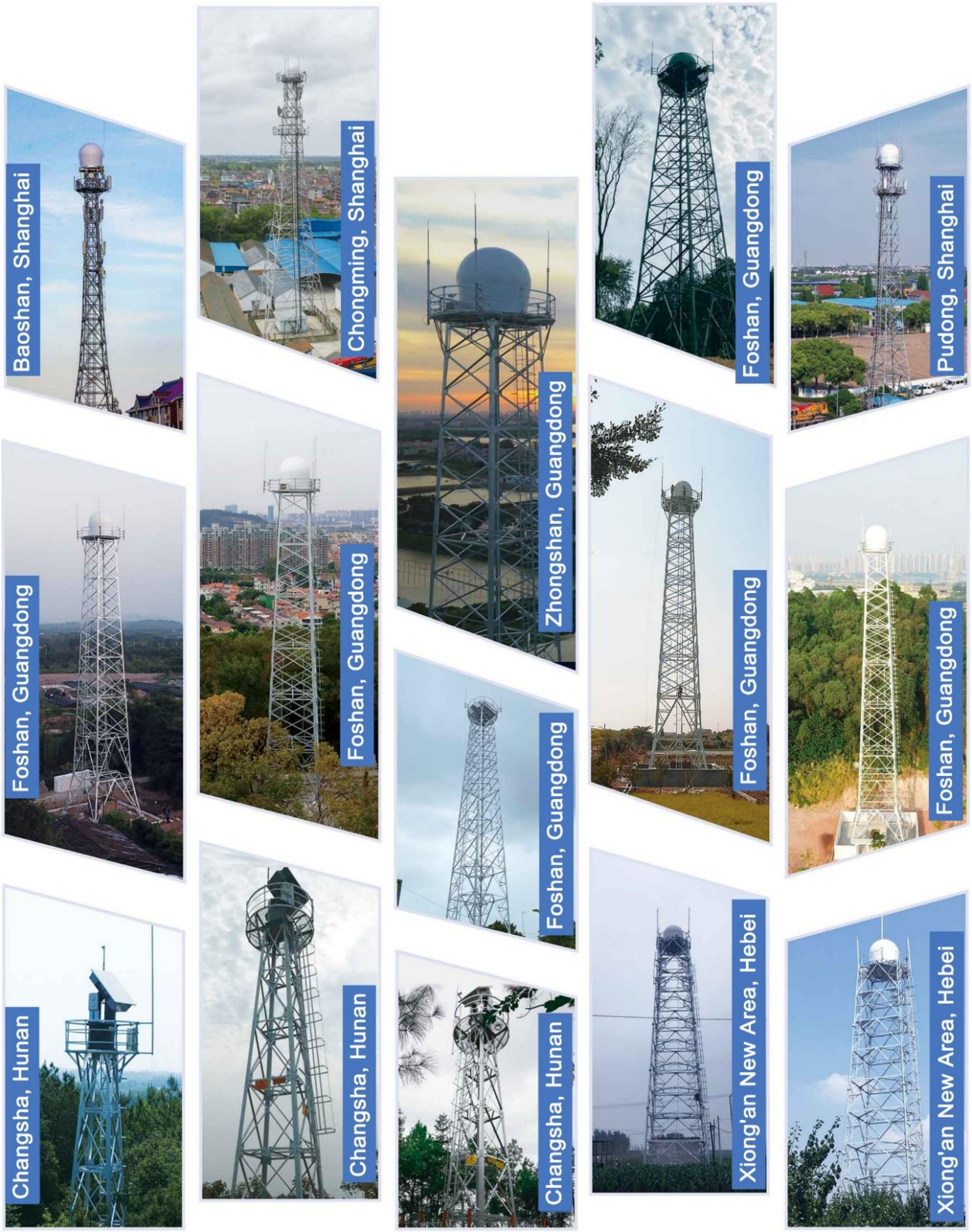

Supplementary Fig. 25 Practical application of the POA/fluoroPOS@silica coating on weather radomes. Photographs of some representative weather radomes with the POA/fluoroPOS@silica coating for preventing rain attenuation in Shanghai, Guangdong, Hebei and Hunan, China.

Supplementary Table 1 Surface chemical composition of the POA/fluoroPOS@silica coating according to the XPS analysis.

Elements	Contents / at.%
C	23.79
O	23.51
F	36.81
Si	15.89

Supplementary Table 2 Comparison of mechanical robustness of various superhydrophobic coatings.

Substrates	Reciprocating abrasion test	Taber abrasion test	Tape-peeling test	Refs.
**ABS**	**After 120 m, CA = 159.9°, SA = 7.5°**	**After 50 cycles, CA = 161.0°, SA = 4.8°**	**After 75 cycles, CA = 156.7°, SA = 9.8°**	**This work**
Glass	After 10 m, CA > 150°, SA = --	--	--	4
Steel	After 10 m, CA = 157°, SA = 6°	--	After 30 cycles, CA = 154°, SA = 8°	5
Iron	After 10 m, CA = 161.8°, SA = --	--	--	6
Steel	After 0.1 m, CA = 150°, SA = --	--	--	7
Silicon	After 2 m, CA > 150°, SA = --	--	After 8 cycles, CA = 140.8°, SA = --	8
Steel	--	After 25 cycles, CA = 138°, SA = --	--	9
Glass	After 3 m, CA = 157.1°, SA = --	--	--	10
Glass	After 5 m, CA = 150°, SA = 13°	--	After 50 cycles, CA = 150°, SA = 12°	11
Ceramic	After 5 m, CA = 146°, SA = 20°	--	--	12
Glass	After 40 m, CA = 150°, SA = 55°	--	--	13
Steel	After 9.65 m, CA = 153°, SA = 7°	--	After 50 cycles, CA = 161°, SA = 5°	14
Glass	--	--	After 60 cycles, CA = 150°, SA = 17°	15
Glass		After 100 cycles, CA > 150°, SA < 10°	After 30 cycles, CA = 154°, SA = 5°	16
Glass	--	After 3000 cycles, CA = 150°, SA < 10°	--	17
Ceramic, metal, glass	After 100 m, CA > 150°, SA < 12°	After 300 cycles, CA = 152°, SA < 12°	After 100 cycles, CA = 154°, SA = 8°	18
Aluminum	After 10 m, CA = 151°, SA = --	--	--	19

"--" means not mentioned.

Supplementary Table 3 Surface chemical composition of the POA/fluoroPOS@silica coating before and after 300 cycles reciprocating abrasion according to the EDS analysis.

Elements	Contents / at. %	
	Before	After
C	57.24	60.77
O	19.63	17.57
F	17.85	16.91
Si	5.28	4.76

Supplementary Table 4 The weather conditions in Yantai, Shandong, P.R. China.

Weather conditions	Values
Annual average temperature	~11.8 °C
Annual average relative humidity	~68%
Annual average rainfall	~651.9 mm
Annual average total solar radiation	~5224.4 MJ m^{-2}

Supplementary Table 5 Comparison of outdoor weather resistance of various superhydrophobic coatings.

Substrates	Outdoor test time	CA after test	SA after test	Refs.
**ABS**	**670 d**	**150.6°**	**29.6°**	**This work**
Glass	90 d	153°	--	[15]
Glass	480 d	152°	--	[20]
Aluminum	120 d	157.8°	7.5°	[21]
Steel	90 d	164°	--	[22]
Silicone rubber	400 d	156°	--	[23]
Glass	10 d	156°	--	[24]
Glass	28 d	160°	2°	[25]
Glass	60 d	150°	10°	[26]

"--" means not mentioned.

Supplementary Table 6 Comparison of the POA/fluoroPOS@silica coating with commercial superhydrophobic coatings.

Product names	CA	SA	Mechanical robustness	Spraying process	Refs.
**POA/fluoroPOS@silica coating**	**165.1°**	**1°**	**After 120 m abrasion CA = 159.9°, SA = 7.5°**	**One-layer spraying**	**This work**
NeverWet	160.0°	3.8°	After 40 m abrasion CA = 130.0°, SA = 19.8°	Two-layer spraying	[27]
Ultra-Ever Dry	154.5°	1°	After 3.6 m abrasion CA = 151.0°, SA = 29°	Two-layer spraying	[28]

Supplementary Table 7 Comparison of applications of superhydrophobic coatings in the field of antennas.

Substrates	CA	SA	Stability	Applications	Performance	Large-scale practical applications	Refs.
**ABS**	**165.1°**	**1°**	**Impalement-resistant, mechanically robust, weather-resistant**	**Preventing rain attenuation of 5G/weather radomes**	**No signal loss after 48 h simulated rainfall**	**Yes**	**This work**
Copper	161°	--	--	Preventing rain attenuation of antennas	No signal loss after 48 h simulated rainfall	--	[29]
Metal surface or nickel foam	--	--	--	Preventing rain attenuation of antennas	No signal loss after placing water droplets	--	[30]
Paper	--	--	--	Anti-water absorption of flexible RF-antennas	No shift in center frequency after soaked in water	--	[31]
PMMA	--	--	--	Anti-eutectic gallium and indium adhesion of liquid metal patch antennas	No visible residue after repeated 7 times	--	[32]
Fabric	164°	--	Mechanically robust	Creating accurate results of patch antenna sensors and protecting users against hazardous droplets	Resonant frequency shift reduced ~8 times	--	[33]

"--" means not mentioned.

Supplementary References

1. Pan, S., Guo, R., Björnmalm, M., Richardson, J. J., Li, L., Peng, C., Bertleff-Zieschang, N., Xu, W.J., Jiang, J.H., & Caruso, F. Coatings super-repellent to ultralow surface tension liquids. *Nat. Mater.* **17**, 1040-1047 (2018).
2. Teisala, H., Geyer, F., Haapanen, J., Juuti, P., Mäkelä, J. M., Vollmer, D., & Butt, H. J. Ultrafast processing of hierarchical nanotexture for a transparent superamphiphobic coating with extremely low roll-off angle and high impalement pressure. *Adv. Mater.* **30**, 201706529 (2018).
3. Zhang, R., Wei, J., Tian, N., Liang, W. & Zhang, J. Facile preparation of robust superamphiphobic coatings on complex substrates via nonsolvent-induced phase separation. *ACS Appl. Mater. Interfaces* **14**, 49047-49058 (2022).
4. Liu, M., Hou, Y., Li, J., Tie, L., Peng, Y. & Guo, Z. Inorganic adhesives for robust, self-healing, superhydrophobic surfaces. *J Mater. Chem. A* **5**, 19297-19305 (2017).
5. Ren, T., Tang, G., Yuan, B., Yang, Y., Yan, Z., Ma, L. & Huang, X. Hexadecyltrimethoxysilane-modified SiO_2 nanoparticle-coated halloysite nanotubes embedded in silicone–acrylic polymer films as durable fluorine-free superhydrophobic coatings. *ACS Appl. Nano Mater.* **3**, 5807-5815 (2020).
6. Zhou, Y., Ma, Y., Sun, Y., Xiong, Z., Qi, C., Zhang, Y. & Liu, Y. Robust superhydrophobic surface based on multiple hybrid coatings for application in corrosion protection. *ACS Appl. Mater. Interfaces* **11**, 6512-6526 (2019).
7. Li, H., Yu, S., Han, X. & Zhao, Y. A stable hierarchical superhydrophobic coating on pipeline steel surface with self-cleaning, anticorrosion, and anti-scaling properties. *Colloid. Surf. A* 503, 43-52 (2016).
8. Zhu, X., Zhou, S., Yan, Q. & Wang, S. Multi-walled carbon nanotubes enhanced superhydrophobic MWCNTs-Co/a-C:H carbon-based film for excellent self-cleaning and

corrosion resistance. *Diam. Relat. Mater.* **86**, 87-97 (2018).

9. Rao, K. S., Yogapriya, R., Raju, K. R. C. S. & Subasri, R. Effect of curing technique on the properties of superhydrophobic coatings. *Trans. Indian Inst. Met.* **74**, 1923-1932 (2021).
10. Chen, B., Qiu, J., Sakai, E., Kanazawa, N., Liang, R. & Feng, H. Robust and superhydrophobic surface modification by a "paint + adhesive" method: applications in self-cleaning after oil contamination and oil-water separation. *ACS Appl. Mater. Interfaces* **8**, 17659-17667 (2016).
11. Sun, Y. & Guo, Z. A scalable, self-healing and hot liquid repelling superamphiphobic spray coating with remarkable mechanochemical robustness for real-life applications. *Nanoscale* **11**, 13853-13862 (2019).
12. Liu, M., Hou, Y., Li, J., Tie, L. & Guo, Z. An all-water-based system for robust superhydrophobic surfaces. *J Colloid Interf. Sci.* **519**, 130-136 (2018).
13. Xue, F., Jia, D., Li, Y. & Jing, X. Facile preparation of a mechanically robust superhydrophobic acrylic polyurethane coating. *J Mater. Chem. A* **3**, 13856-13863 (2015).
14. Wang, N., Lu, Y., Xiong, D., Carmalt, C. J. & Parkin, I. P. Designing durable and flexible superhydrophobic coatings and its application in oil purification. *J.Mater. Chem. A* **4**, 4107-4116 (2016).
15. Li, K., Zeng, X., Li, H. & Lai, X. Fabrication and characterization of stable superhydrophobic fluorinated-polyacrylate/silica hybrid coating. *Appl. Surf. Sci.* **298**, 214-220 (2014).
16. Peng, C., Chen, Z. & Tiwari, M. K. All-organic superhydrophobic coatings with mechanochemical robustness and liquid impalement resistance. *Nat. Mater.* **17**, 355-360 (2018).
17. Zhang, H., Bu, X., Li, W., Cui, M., Ji, X., Tao, F., Gai, L., Jiang, H., Liu, L. & Wang, Z. A skin-inspired design integrating mechano-chemical-thermal robustness into superhydrophobic coatings. *Adv. Mater.* **34**, e2203792 (2022).

18. Wang, D., Sun, Q., Hokkanen, M. J., Zhang, C., Lin, F. Y., Liu, Q., Zhu, S. P., Zhou, T., Chang, Q., He, B., Zhou, Q., Chen, L., Wang, Z., Ras, R. H. A. & Deng, X. Design of robust superhydrophobic surfaces. *Nature* **582**, 55-59 (2020).

19. Li, Y., Ma, W., Kwon, Y. S., Li, W., Yao, S. & Huang, B. Solar deicing nanocoatings adaptive to overhead power lines. *Adv. Funct. Mater.* **32**, 2113297 (2022).

20. Tan, X., Wang, Y., Huang, Z., Sabin, S., Xiao, T., Jiang, L. & Chen, X. Facile fabrication of a mechanical, chemical, thermal, and long-term outdoor durable fluorine-free superhydrophobic coating. *Adv. Mater. Interfaces* **8**, 2002209 (2021).

21. Zhao, Z., Wang, H., Liu, Z., Zhang, X., Zhang, W., Chen, X. & Zhu, Y. Durable fluorine-free superhydrophobic polyethersulfone (PES) composite coating with uniquely weathering stability, anti-corrosion and wear-resistance. *Prog. Org. Coat.* **127**, 16-26 (2019).

22. Cho, E. C., Chang-Jian, C. W., Chen, H. C., Chuang, K. S., Zheng, J. H., Hsiao, Y. S., Lee, K. C. & Huang, J. H. Robust multifunctional superhydrophobic coatings with enhanced water/oil separation, self-cleaning, anti-corrosion, and anti-biological adhesion. *Chem. Eng. J.* **314**, 347-357 (2017).

23. Oh, S., Shim, J., Seo, D., Shim, M., Han, S., Lee, C. & Nam, Y. Organic/inorganic hybrid cerium oxide-based superhydrophobic surface with enhanced weather resistance and self-recovery. *Prog. Org. Coat.* **170**, 106998 (2022).

24. Li, J., Zhou, L., Yang, N., Gao, C. & Zheng, Y. Robust superhydrophobic coatings with micro- and nano-composite morphology. *RSC Adv.* **7**, 44234-44238 (2017).

25. Lyu, J., Wu, B., Wu, N., Peng, C., Yang, J., Meng, Y. & Xing, S. Green preparation of transparent superhydrophobic coatings with persistent dynamic impact resistance for outdoor applications. *Chem. Eng. J.* **404**, 126456 (2021).

26. Liu, Y., Tan, X., Li, X., Xiao, T., Jiang, L., Nie, S., Song, J. & Chen, X. Eco-friendly fabrication of transparent superhydrophobic coating with excellent mechanical robustness,

chemical stability, and long-term outdoor durability. *Langmuir* **38**, 12881-12893 (2022).

27 Zhi, D., Wang, H., Jiang, D., Parkin, I. P. & Zhang, X. Reactive silica nanoparticles turn epoxy coating from hydrophilic to super-robust superhydrophobic. *RSC Adv.* **9**, 12547-12554 (2019).

28 Wang, L., Yang, J., Zhu, Y., Li, Z., Sheng, T., Hu, Y. M., & Yang, D. A study of the mechanical and chemical durability of Ultra-Ever Dry Superhydrophobic coating on low carbon steel surface. *Colloids Surf. A* **497**, 16-27 (2016).

29. Ge, X. L., Yang, J. H., Ren, H., Qin, Z. J., Chen, Q. D., Han, D. D., Zhang, Y. L., Xu, S. & Sun, H. B. Commercial-printed-circuitry-compatible self-superhydrophobic antennas based on laser direct writing. *Prog. Electromagn. Res.* **176**, 45-53 (2023).

30. Ge, X. L., Han, D. D. & Xu, S. Laser direct writing based superhydrophobic antenna. *IEEE Conf. Antenna Meas. Appl.* 10002652 (2022).

31. Foroughian, F., Ghahremani, A., Fathy, A. E. & Simpson, J. Flexible RF-antennas coated by a superhydrophobic paint for minimal water absorption. *IEEE Int. Symp. Antennas Propag.* 1947-1948 (2016).

32. Bharambe, V., Adams, J. J., Joshipura, I. D., Ayers, H. R. & D.Dickey, M. Reversibly reconfigurable liquid metal patch antenna using a superhydrophobic spray-coating. *IEEE Trans. Antennas Propag.* 287-288 (2018).

33. Kazemi, K. K., Zarifi, T., Mohseni, M., Narang, R., Golovin, K. & Zarifi, M. H. Smart superhydrophobic textiles utilizing a long-range antenna sensor for hazardous aqueous droplet detection plus prevention. *ACS Appl. Mater. Interfaces* **13**, 34877-34888 (2021).
