# Supplementary material for: Durable superhydrophobic coatings for prevention of rain attenuation of 5G/weather radomes
Source: Nat Commun. 2023 May 19;14:2862. doi: 10.1038/s41467-023-38678-0 (PMC10198997; doi:10.1038/s41467-023-38678-0)
Supplement: Supplementary file 3 — Description of Additional Supplementary Information [file 41467_2023_38678_MOESM3_ESM.pdf]

Description of Additional Supplementary Information

Title: Supplementary Movie 1

Description: Impact/bounce of a 10 µL water droplet on the POA/fluoroPOS@silica coating.

Title: Supplementary Movie 2

Description: Rolling of water droplets on the POA/fluoroPOS@silica coating after 120 s high pressure water jetting at 50 kPa or after 20 h simulated rainfall.

Title: Supplementary Movie 3

Description: Rolling of water droplets on the POA/fluoroPOS@silica coating after 300 cycles reciprocating abrasion, 50 cycles Taber abrasion or 75 cycles tape-peeling.

Title: Supplementary Movie 4

Description: Impact/bounce of a 10 µL water droplet released from 100 cm height on the POA/fluoroPOS@silica coating after 300 cycles reciprocating abrasion.

Title: Supplementary Movie 5

Description: Mechanical robustness tests of the POA/fluoroPOS@silica coating by intensive hand rub under water and abrasion with steel wool.

Title: Supplementary Movie 6

Description: Water droplet freezing process on the ABS plate and POA/fluoroPOS@silica coated ABS plate.

Title: Supplementary Movie 7

Description: Rolling of water droplets on the colorful POA/fluoroPOS@silica coatings.

Title: Supplementary Movie 8

Description: Rolling of raindrops on the POA/fluoroPOS@silica coated 5G radomes.

Supplementary Movie 9

Description: Spraying the POA/fluoroPOS@silica coating on a weather radome and its superhydrophobicity to bulk water.

Title: Supplementary Movie 10

Description:Rolling of water droplets on the POA/fluoroPOS@silica coated weather radome after 1 year of practical application.
